# Cryptogenic stroke in women: impact of insertable cardiac monitoring in the STROKEWISE cohort study

**DOI:** 10.1093/europace/euag056

**Published:** 2026-03-23

**Authors:** Jannicke Koldéus-Falch, Bente Thommessen, Antje Sundseth, Loreta Skrebelyte-Strøm, Halvor Næss, Owen M Truscott Thomas, Ole Morten Rønning

**Affiliations:** Department of Neurology, Akershus University Hospital, PO Box 1000, 1478 Lørenskog, Norway; Institute of Clinical Medicine, University of Oslo, PO Box 1171 Blindern, 0318 Oslo, Norway; Department of Neurology, Akershus University Hospital, PO Box 1000, 1478 Lørenskog, Norway; Department of Neurology, Akershus University Hospital, PO Box 1000, 1478 Lørenskog, Norway; Department of Neurology, Akershus University Hospital, PO Box 1000, 1478 Lørenskog, Norway; Department of Cardiology, Akershus University Hospital, Lørenskog, Norway; Department of Neurology, Haukeland University Hospital, Bergen, Norway; Health Services Research (HØKH), Akershus University Hospital, Lørenskog, Norway; Department of Neurology, Akershus University Hospital, PO Box 1000, 1478 Lørenskog, Norway; Institute of Clinical Medicine, University of Oslo, PO Box 1171 Blindern, 0318 Oslo, Norway

**Keywords:** Stroke, Atrial fibrillation, Prolonged cardiac monitoring, Implantable loop recorder, Women, Anticoagulation

## Abstract

**Aims:**

The clinical value of insertable cardiac monitoring (ICM) for detecting asymptomatic atrial fibrillation (AF) and guiding anticoagulation in patients with cryptogenic stroke remains uncertain. This study aimed to evaluate the association between ICM-detected AF and recurrent stroke, mortality, and major bleeding in women.

**Methods and results:**

We consecutively compared women with cryptogenic stroke who received an ICM at Akershus University Hospital (2016–23), with a control group from Haukeland University Hospital with standard non-invasive follow-up. Participants from both hospitals had complete data in the Norwegian Stroke Registry. Primary outcomes were AF detection and recurrent stroke at 1 and 2 years; secondary outcomes included mortality, composite stroke and mortality, ischaemic cardiovascular events, oral anticoagulation initiation, and major bleeding. Multivariable logistic regression analysis was used for binary outcomes and Cox proportional hazards models for time-to-event outcomes, both adjusted for prespecified vascular risk factors. Among 475 women (mean age 73 ± 12 years; ICM *n* = 262), AF was detected in 36% in the ICM group vs. 9% in in the control group (OR 6.9, 95% CI 3.7–13.5; *P* < 0.001). No significant differences were observed in recurrent stroke (HR 1.8, 95% CI 0.78–4.36; *P* = 0.17), mortality (HR 0.88, 95% CI 0.58–1.58; *P* = 0.88), or the composite outcome (HR 0.96, 95% CI 0.6–1.5; *P* = 0.85) with a median follow-up of 42 months (IQR 15–66). However, fixed-time analyses showed significantly lower 2-year mortality with ICM (OR 0.40, 95% CI 0.16–0.94; *P* = 0.036) and fewer bleeding events (OR 0.19, 95% CI 0.06–0.52; *P* = 0.002). Age and troponin T were consistent independent predictors of adverse outcomes.

**Conclusion:**

ICM increased AF detection and enabled safe anticoagulation in women with acute cryptogenic stroke. Although stroke recurrence did not differ significantly, signals of lower mortality and major bleeding support prolonged monitoring to optimize secondary prevention.

**ClinicalTrials.gov Identifier:**

NCT07194811, https://clinicaltrials.gov/study/NCT07194811

## Graphical Abstract

**Graphical Abstract:**
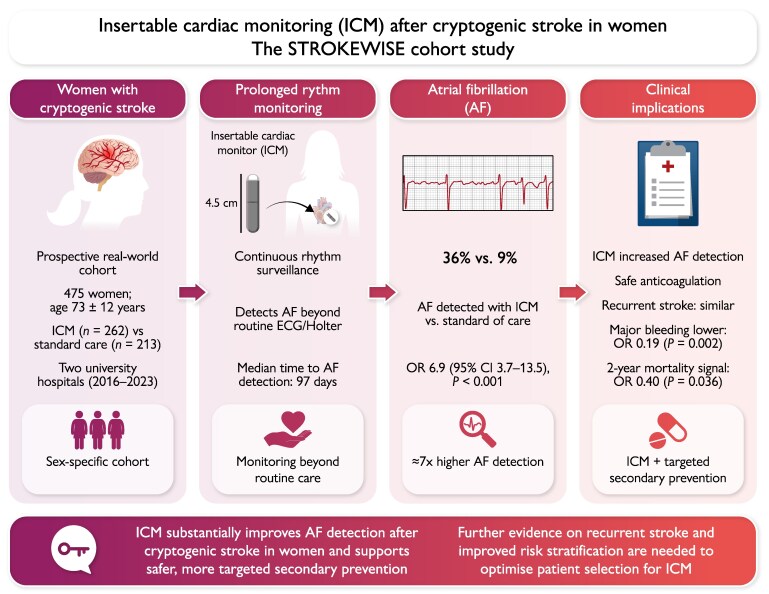

What’s new?

- This large, prospective real-world study is the first to focus exclusively on women with cryptogenic stroke undergoing insertable cardiac monitoring (ICM), compared with standard of care.
- Prolonged monitoring with ICM increased AF detection nearly seven-fold, but recurrent ischaemic stroke rates remained similar between groups, likely reflecting substantial anticoagulant use in both cohorts.
- AF was identified in only about one-third of women, underscoring the need to distinguish patients with AF from those with other underlying mechanisms.
- Prolonged monitoring with ICM was required to identify this subgroup, as most AF episodes occurred beyond routine monitoring periods.
- Oral anticoagulation initiated after documented AF was safe, with significantly fewer major bleeding events in the ICM group.
- These findings address a major evidence gap in women’s stroke care by providing sex-specific data on prolonged rhythm monitoring and outcomes after cryptogenic stroke.

## Introduction

Women are at higher risk of atrial fibrillation (AF)-related stroke, experience greater stroke severity, and have worse outcomes compared to men.^1,2^ Social factors, such as living alone in older age, may further impair recovery and quality of life.^3,4^ These sex differences are only partly explained by age and may reflect distinct vascular risk profiles, post-menopausal hormonal changes, prothrombotic mechanisms,^5,6^ and disparities in access to diagnostic evaluation and treatment.^7^

Cryptogenic stroke, including its subgroup embolic stroke of undetermined source (ESUS), represents up to one-third of ischaemic strokes.^8^ Device-detected AF (DDAF) is commonly asymptomatic after cryptogenic stroke and often missed by short-term ECG monitoring,^9^ and a meta-analysis reported similar thromboembolic risk and mortality in asymptomatic and symptomatic AF.^10^ Insertable cardiac monitors (ICMs) provide continuous rhythm monitoring, outperform conventional methods in detecting subclinical AF, and allow quantification of AF burden.^11,12^ However, the clinical benefit of oral anticoagulation (OAC) after DDAF remains uncertain, although a meta-analysis concluded that OAC is superior to placebo or aspirin in reducing stroke in high-risk patients with DDAF, at the cost of increased bleeding.^13^ Of note, only ∼10% of participants in these trials had a prior stroke or transient ischaemic attack (TIA)—typically a remote stroke (70% > 1 year)—and fewer than 37% were women.^14^

Significant knowledge gaps remain regarding sex-specific risk factors and outcomes in stroke and AF. Women remain underrepresented in cardiovascular trials, including studies on cardiac monitoring^15^ and secondary stroke prevention.^16^ Consequently, evidence in women with acute cryptogenic stroke is limited regarding the clinical benefit of AF detection with ICM, and the efficacy and safety of subsequent anticoagulation.

The aim of the study was to assess the association between ICM use and outcomes compared with standard non-invasive follow-up in women after acute cryptogenic stroke. Primary outcomes were AF detection and recurrent stroke.

## Methods

### Study design and patient selection

STROKEWISE (STROKE of unknown cause in Women: the Impact of long-term heart monitoring on Stroke recurrencE, ClinicalTrials.gov Identifier NCT07194811) is a prospective, observational, real-world cohort study at two university hospitals in Norway with comprehensive stroke units, standardized diagnostic protocols, and aetiological stroke classification. Two consecutive cohorts of patients with acute cryptogenic stroke were prospectively recorded between 2016 and 2023. At Akershus University Hospital (AUH), patients were offered ICM, while a historical control cohort from Haukeland University Hospital (HUH) comprising all admitted cryptogenic stroke patients in the same study period, received standard non-invasive post-stroke monitoring without systematic prolonged follow-up thereafter. Clinical data were linked with national health registries. The study is reported in accordance with the STROBE guidelines.

**Figure 1:**
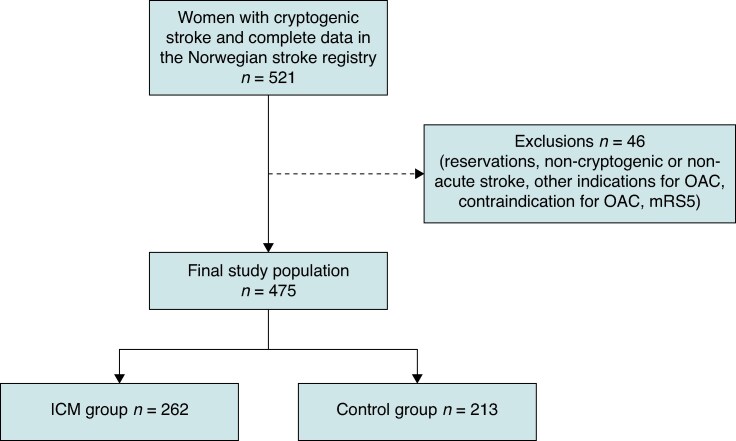
Flowchart of the study population.

Eligible participants for this analysis were women aged ≥18 years with acute cryptogenic stroke defined by TOAST criteria (Trial of ORG 10172 in Acute Stroke Treatment classification).^17^ Exclusion criteria were prior AF, ongoing or contraindications to OAC, stroke of known aetiology, or severe pre-stroke disability modified Rankin scale (mRS) = 5.^18^ We excluded patients with AF detected before discharge (classified as cardioembolic stroke) or incomplete Norwegian Stroke Registry (NSR) data (*Figure 1*).

### Data collection and outcomes

Baseline characteristics, demographics, stroke severity (National Institutes of Health Stroke Scale, NIHSS), disability (mRS), and laboratory parameters were obtained from hospital quality registries, electronic records, and national health registries. Endpoint events, diagnoses, and OAC use during follow-up were obtained through linkage to the NSR, the national patient registry (NPR), and the Norwegian Prescription Database (NorPD), all validated for epidemiological research.^19,20,21^ Analyses were performed on deidentified, linked data.

Acute and recurrent stroke events were identified using ICD-10 codes I61, I63, and I64 (International Classification of Diseases 10th Revision). Symptomatic intracranial haemorrhage (ICH; I61) was defined according to ECASS III criteria.^22^ From the NPR, we captured admissions for ischaemic heart disease (I20–I25), AF (I48), and gastrointestinal bleeding (K92). OAC exposure (dabigatran, rivaroxaban, apixaban, edoxaban, warfarin) was obtained from dispensed prescriptions in NorPD. The index date was admission, and all patients at both centres underwent a standardized aetiological workup with stroke diagnosis confirmed by two neurologists and TOAST classification applied after cerebral CT and/or MRI, vascular imaging, 12-lead ECG, Holter or telemetry, and laboratory testing; echocardiography was performed when indicated. In the control cohort, further rhythm assessment after discharge was at the discretion of treating physicians (e.g. additional Holter, event recorders, or ECG in case of symptoms), but there was no protocol-mandated long-term rhythm monitoring. ICM patients received a subcutaneous device (Reveal LINQ I or II, Medtronic, Minneapolis, MN, USA) before discharge or within 30 days after the index event. The diagnostic workup for this cohort has been described previously.^23^ AF episodes were detected via remote monitoring (MyCareLink), defined as an irregular rhythm without visible P-waves lasting ≥30 s and confirmed by two cardiologists, blinded for baseline data. All devices were connected to remote monitoring with automated nightly transmissions of stored episodes and alerts. Device data were reviewed at least once per week, and additional interrogations were performed in response to alerts or patient-activated events. In the control cohort, the specific mode of AF detection was not captured in the national registries. Antiplatelet therapy was switched to OAC (mainly direct OAC) upon AF detection within days and after individual assessment. Controls received OAC empirically at discharge at the clinician’s discretion; otherwise, antiplatelet therapy was prescribed. Thus, all cryptogenic stroke patients in both cohorts received antiplatelet therapy unless OAC was initiated. No patients in the ICM cohort were discharged on empirical OAC without documented AF.

Primary outcomes were AF detection and recurrent ischaemic stroke at 30 days, 1 year, and 2 years. Secondary outcomes included all-cause mortality, a composite of any recurrent stroke and mortality, ischaemic cardiovascular events, OAC use, and major bleeding events including gastrointestinal bleeding and symptomatic ICH.

## Statistical analysis

A statistical analysis plan was developed prior to accessed data. Descriptive statistics characterized the cohort. Continuous variables were presented as mean ± SD or median (IQR); categorical variables were presented as counts (%), compared using *χ*^2^, Fisher’s exact test, *t*-test, or Wilcoxon rank-sum as appropriate. Logistic regression estimated odds ratios (ORs) with 95% confidence intervals (CIs) for binary outcomes; Cox models assessed time-to-event outcomes with proportional hazards assumptions tested. Analyses were censored at AF detection, recurrent stroke, death, or end of follow-up. Multivariable models were adjusted for age, diabetes, hypertension, prior stroke, prior ischaemic heart disease, low-density lipoprotein (LDL), and log-transformed high-sensitivity cardiac troponin T (hs-cTnT). Sensitivity analysis for missing values was consistent with the main findings; no imputation was performed. A two-sided *P*-value of <0.05 was considered statistically significant. Analyses used STATA SE 18.0 and R 3.6.

## Results

We included 475 women (ICM *n* = 262, control *n* = 213) with a median follow-up of 42 months (IQR 15–66). The mean age was 73 ± 12 years. Baseline characteristics are shown in *Table 1*. Groups were generally well balanced, although the ICM group had higher rates of prior stroke and TIA.

**Table 1:** Baseline characteristics of women with cryptogenic stroke

	ICM group (*n* = 262)	Control group (*n* = 213)	Total (*n* = 475)
**Demography**			
Age, mean (SD)	72 (±11)	74 (±13)	73 (±12)
Age, median (IQR)	74 (67–79)	76 (67–83)	75 (67–81)
<65 years	50 (19%)	43 (20%)	93 (20%)
65–74 years	89 (34%)	52 (24%)	141 (30%)
75+ years	123 (47%)	118 (55%)	241 (51%)
Living alone	119 (45%)	96 (45%)	215 (45%)
**Comorbidities**			
Prior hypertension	167 (64%)	24 (58%)	291 (61%)
Prior diabetes mellitus	35 (13%)	32 (15%)	67 (14%)
Prior hypercholesterolaemia	95 (36%)	47 (22%)	142 (30%)
Current smoking	53 (20%)	41 (20%)	94 (20%)
Prior smoking	66 (25%)	58 (27%)	124 (26%)
Prior ischaemic stroke	44 (17%)	18 (9%)	62 (13%)
Prior myocardial infarction	17 (7%)	9 (4%)	26 (6%)
Prior TIA	22 (8%)	8 (4%)	30 (6%)
**NIHSS**			
Mean (SD)	3.3 (4.5)	5.0 (5.0)	4.1 (4.8)
Median (IQR)	2.0 (0–4)	3 (2–7)	2.0 (1–6)
0–4	203 (77%)	124 (58%)	327 (69%)
5–9	32 (12%)	58 (27%)	90 (19%)
≥10	27 (10%)	31 (15%)	58 (12%)
**Pre-stroke mRS**			
Mean (SD)	0.21 (0.6)	0.26 (0.7)	0.24 (0.7)
Median (IQR)	0 (0–1)	0 (0–1)	0 (0–1)
mRS 0–2	256 (98%)	186 (87%)	442 (93%)
mRS 3–4	6 (2%)	27 (13%)	33 (7%)
**Imaging and treatment**			
CT only	100 (38%)	11 (5%)	111 (23%)
CT and MRI	162 (62%)	202 (95%)	364 (77%)
Thrombolysis	63 (24%)	76 (36%)	139 (29%)
Thrombectomy	58 (22%)	20 (9%)	78 (16%)
**Biomarkers**	*n* = 239	*n* = 188	
Troponin T (ng/L) median (IQR)	12 (8–23)	4 (0.1–11)	9 (4–18)
	*n* = 226	*n* = 195	
LDL (mmol/L) median (IQR)	2.6 (1.9–3.3)	3.5 (2.7–4.3)	3.0 (2.1–3.8)
**ICM (days after index)**			
Median (IQR)	4 (1–7)		
Mean (SD)	4.6 (3.9)		

Mean (SD), median (IQR), or *n* (%) unless otherwise stated.

NIHSS, National Institutes of Health Stroke Scale; mRS, modified Rankin scale; ICM, insertable cardiac monitor; TIA, transient ischaemic attack; LDL, low-density lipoprotein.

### Primary outcomes

AF was detected in 93/262 (36%) ICM patients and 19/213 (9%) controls (OR 6.9, 95% CI 3.7–13.5) over total follow-up. Detection in the ICM group increased over time, reaching 11% at 30 days, 32% at 1 year, and 34% at 2 years. Median time to detection was 97 days (IQR 26–217). The duration of the first AF episode was <6 min in 32%, ≥6 min to <6 h in 33%, 6–24 h in 20%, and >24 h in 8% of patients (7% missing).

Recurrent ischaemic stroke occurred at an annual rate of ∼3% and did not differ significantly between groups (Cox HR 1.8, 95% CI 0.8–4.4; 365-day OR 0.92, 95% CI 0.25–3.6). Of note, 26% of controls were discharged on empirical OAC. Recurrent stroke rates were comparable at each time point: 0.4% vs. 0% at 30 days, 3.1% vs. 2.8% at 1 year, and 3.4% vs. 4.2% at 2 years (ICM vs. control). Cumulatively, 7% in each group experienced recurrent stroke. AF was present in 9/32 patients (28%) with recurrent stroke, with similar proportions across groups (33% vs. 21%).

The main clinical outcomes by groups are presented in *Tables 2* and *3*.

**Table 2:** Primary and secondary outcomes at 2 years (fixed-time analysis) and total follow-up. Adjusted odds ratios (95% CI) from multivariable logistic regression models unless indicated

Primary and secondary outcomes	ICM group (*n* = 262)	Control group (*n* = 213)	OR (95% CI)	*P*-value
**Fixed-time outcomes (2 years)**				
Recurrent ischaemic stroke	9 (3.4%)	9 (4.2%)	0.78 (0.24–2.54)	0.678
Mortality (all-cause)	10 (3.8%)	23 (10.8%)	0.40 (0.16–0.94)	**0**.**036**
Composite any recurrent stroke and mortality	20 (7.6%)	35 (16%)	0.37 (0.17–0.77)	**0**.**008**
**Outcomes over total follow-up**				
Atrial fibrillation	93 (36%)	19 (9%)	6.90 (3.7–13.55)	**<0**.**0001**
Oral anticoagulation use	109 (42%)	75 (35%)	1.40 (0.87–2.24)	0.175
Cardiovascular ischaemic events	58 (22%)	41 (19%)	0.87 (0.47–1.58)	0.637
Major bleeding events	8 (3%)	20 (9%)	0.19 (0.06–0.52)	**0**.**002**
GI bleeding	6 (2%)	14 (7%)	0.33 (0.10–0.95)^a^	**0**.**021**
Symptomatic ICH	2 (0.8%)	6 (2.8%)	0.27 (0.03–1.51)^a^	0.084

OR, odds ratio; CI, confidence interval; ICH, intracranial haemorrhage; GI, gastrointestinal

^a^Unadjusted.

**Table 3:** Primary and secondary outcomes (time-to-event analyses). Adjusted hazard ratios (95% CI) from multivariable Cox regression models

Primary and secondary outcomes	ICM group (*n* = 262)	Control group (*n* = 213)	HR (95% CI)	*P*-value
Recurrent ischaemic stroke	18 (7%)	14 (7%)	1.84 (0.78–4.36)	0.17
Mortality (all-cause)	39 (15%)	68 (32%)	0.96 (0.58–1.58)	0.88
Composite any recurrent stroke and mortality	50 (19%)	78 (37%)	0.96 (0.61–1.49)	0.85

Counts (*n*, %) reflect cumulative incidence over total follow-up.

HR, hazard ratio; CI, confidence interval.

To further explore potential risk factors beyond AF, we performed additional exploratory analyses (see Supplementary material online, *Tables S1*–*S2*). In univariable analyses, recurrent stroke was associated with hypertension and higher creatinine levels, with a trend towards more frequent prior ischaemic strokes, whereas diabetes, dyslipidaemia, smoking, prior TIA, and age were not significantly associated. In multivariable logistic regression adjusting for AF and age, hypertension remained independently associated with recurrent stroke, while AF was not.

### Secondary outcomes

Mortality consistently favoured the ICM group, with lower proportions at 30 days, 1 year, and 2 years (0% vs. 0.9%, 1.5% vs. 5.6%, and 3.8% vs. 10.8%, respectively). At 2 years, fixed-time analysis showed significantly lower mortality with ICM (OR 0.40, 95% CI 0.16–0.94), although no significant difference was observed in Cox analysis (HR 0.96, 95% CI 0.58–1.58). The composite of any recurrent stroke and mortality showed a similar pattern, with lower 2-year risk in the ICM group (7.6% vs. 16%; OR 0.37, 95% CI 0.17–0.77), but no difference in time-to-event analysis (HR 1.0, 95% CI 0.6–1.5). OAC use was more frequent in the ICM group (42% vs. 35%), although not significant after adjustment. Ischaemic cardiovascular events (cardiac events and stroke) did not differ significantly between groups.

Major bleeding events (gastrointestinal bleeding and symptomatic ICH) were less frequent in the ICM group compared with the control group (3% vs. 9%), supported by a significant association (adjusted OR 0.19, 95% CI 0.06–0.52), while symptomatic ICH remained rare in both cohorts (0.8% vs. 2.8%). There was no significant association between AF and ICH (2.7% vs. 1.4%), but seven of eight ICH events occurred in patients receiving OAC.

Bleeding complications were consistently more frequent among patients receiving OAC, particularly in the control group, where empirical initiation of OAC at discharge was practiced. In the control group, gastrointestinal bleeding occurred in 9.3% of OAC-treated patients compared with 6.6% in the overall control cohort, whereas the corresponding proportions in the ICM group were 2.8% and 2.3%. Similarly, ICH occurred more frequently in OAC-treated patients in the control group (6.7%) than overall in that group (2.8%), and discharge OAC was associated with the highest observed risk of ICH [4/55 (7.3%) vs. 2/158 (1.3%); *P* = 0.020], whereas lower rates were observed in the ICM group (1.8% among OAC-treated patients and 0.8% overall).

These findings indicate that bleeding risk was higher among patients receiving OAC—most prominently in the control group, where anticoagulation was not targeted to documented AF.

Older age and higher hs-cTnT independently predicted AF detection, recurrent stroke, and mortality, although the study was not designed to assess individual predictors.

As exploratory, unadjusted analyses, we cross-tabulated recurrent stroke, major bleeding, and mortality by hospital and OAC status and additionally examined recurrent stroke in the subgroup of patients who did not receive OAC during follow-up. Detailed results are presented in Supplementary material online, *Tables S3* and *S4* and were broadly consistent with the main findings, although based on a few events.

## Discussion

This study of women with acute cryptogenic stroke identified several clinically important findings. First, ICM substantially increased AF detection compared to standard non-invasive follow-up; the odds were seven-fold, and most detections occurred within the first year. Second, recurrent ischaemic stroke rates were similar between groups, and the time-to-event analyses were neutral. The annual recurrence risk of ≈3% was low, and only about one in four events occurred in patients with documented AF, suggesting competing stroke aetiologies. Third, mortality and the composite of stroke and death consistently favoured the ICM group, with significant differences at 2 years in fixed-time analyses, although not in time-to-event models and should therefore be interpreted cautiously. Fourth, safety outcomes favoured an ICM-guided approach, with significantly fewer major bleeding events despite greater overall anticoagulation use.

Taken together, these findings highlight two key clinical messages. First, prolonged monitoring markedly improves AF detection in women with cryptogenic stroke, identifying a substantial proportion of AF missed by short-term strategies. The median time to AF detection (97 days) underscores that this arrhythmia often occurs after completion of standard short-term monitoring, which is clinically relevant given uncertainties around the temporal relationship between AF and stroke.^24^ Second, targeted anticoagulation after verified AF appears safer than empirical treatment. The lack of association between AF and ICH, combined with the clear link between empirical anticoagulation and bleeding risk, emphasizes the importance of individualized strategies—treating AF when detected, particularly in older women who face both high thromboembolic risk and bleeding concerns.

Our observations align with the hypothesis that women—who experience a disproportionate burden of AF-related stroke and have historically been underdiagnosed and undertreated—may benefit from prolonged rhythm monitoring linking diagnosis to treatment. However, we did not observe a difference in recurrent stroke between the groups, and several factors may explain this finding. Importantly, a quarter of the control group was discharged on empirical OAC despite no documented AF, in patients considered at high thromboembolic risk, and reflecting real-world clinical practice during the study period. This practice may have reduced observable differences in anticoagulation exposure between groups and could have attenuated potential differences in stroke recurrence, while possibly contributing to an increased risk of major bleeding, particularly among patients anticoagulated without documented AF. Although symptomatic ICH remained rare, the higher frequency of major bleeding in the control cohort—where anticoagulation was not consistently targeted—reinforces that OAC should be initiated after verified AF rather than empirically. Of note, AF was present in only a minority of patients with recurrent stroke, supporting multifactorial mechanisms. Hypertension emerged as an independent predictor of recurrent stroke, even after adjustment for AF and age, underscoring the contribution of overall vascular burden and suggesting that non-embolic aetiologies account for a substantial share of recurrent events. Competing risks, the low stroke event rate, limited statistical power, and the relatively short follow-up may also have reduced our ability to detect differences in stroke recurrence between groups. The signal towards lower mortality and composite outcomes in the ICM group may indicate broader cardiovascular benefits of early AF detection and targeted anticoagulation, although these findings should be interpreted cautiously.

Secondary stroke prevention depends on clarifying the underlying mechanism of the index event. A recent pooled individual-patient meta-analysis (ANTARCTICA) indicates that DDAF is more frequent, occurs earlier, and is of longer duration in cryptogenic stroke compared with non-cryptogenic stroke, supporting a causal relationship in approximately half of patients.^25^ Antiplatelet therapy is recommended for secondary prevention after cryptogenic stroke^26^; however, it is inadequate when AF is present. As AF is identified in only about one-third of patients, indiscriminate anticoagulation cannot be justified. Accurate identification of this subgroup is therefore important, and prolonged rhythm monitoring with ICM represents a reliable and safe approach to guide secondary prevention in patients with DDAF, with anticoagulation decisions made on an individual basis.

Patients in whom AF is detected after stroke (AFDAS) may have a lower risk of recurrence and a more favourable cardiovascular profile than those with known AF.^27,28^ Consistent with this, the estimated annual recurrent stroke risk of ∼3% lies at the lower end of published reports in cryptogenic stroke.^29^ This likely reflects a combination of substantial OAC use in both groups and optimized management of vascular risk factors.

Our AF detection rates, timing, and safety outcomes align with prior randomized and observational studies,^9,23,30,31^ which consistently show that prolonged monitoring outperforms short-term strategies. In contrast, the modest between-group difference in OAC use observed in our study likely reflects a site-specific practice pattern, including empirical OAC use in the control cohort. The favourable bleeding profile observed with ICM-guided management adds nuance to previous neutral or negative ESUS trials,^32^ reinforcing that anticoagulation should be guided by confirmed AF rather than by embolic stroke phenotype alone.

Current guidelines recommend prolonged rhythm monitoring after stroke of undetermined origin, and our findings align with and support their implementation in clinical practice.^33,34,35^ Nevertheless, post-stroke use of ICM and management of DDAF vary substantially across centres and countries, as highlighted in a recent EHRA survey,^36^ underscoring the lack of consensus and the need for harmonized, evidence-based strategies.

This study’s strengths include its exclusive focus on women with cryptogenic stroke, addressing an important evidence gap regarding sex-specific data, and its prospective real-world design using high-quality national registry data. Limitations include the non-randomized design, which introduces potential residual confounding and site-specific practice differences (empirical OAC), and baseline imbalances may have influenced outcomes despite adjustment. Registry-based coding carries a risk of misclassification, incomplete AF burden data or symptom status for AF, and lack of cause-of-death information. Generalizability is most applicable to similar high-income health systems. Although the cohort was substantial, the number of events was modest, limiting statistical power for some outcomes.

Regardless of sex, important knowledge gaps remain regarding clinical outcomes and the added benefit of ICM compared with standard monitoring, particularly how AF burden and subtype influence thromboembolic risk and anticoagulation safety.^37,38,39,40^ Cryptogenic stroke is a heterogeneous group with multiple non-AF-related mechanisms, underscoring the need to improve patient selection for AF detection and to assess overall thromboembolic risk beyond AF alone. Future studies should integrate blood-based (biochemical and genetic), cardiological, and neuroimaging markers to enhance risk stratification, both to identify which patients are most likely to benefit from ICM and to guide complementary preventive strategies beyond rhythm monitoring in this population.^38^

## Conclusions

ICM markedly increased AF detection in women with cryptogenic stroke and enabled safe, targeted initiation of anticoagulation. Although we did not observe a significant difference in stroke recurrence, signals of lower mortality and major bleeding were seen with ICM-guided management. These findings support considering ICM as a safe strategy to guide secondary prevention after acute cryptogenic stroke. Larger multicentre randomized trials are needed to clarify the impact of ICM on recurrent stroke and to refine risk stratification for optimal patient selection.

## Supplementary Material

euag056_Supplementary_Data

## Data Availability

Deidentified data underlying this article are not publicly available due to Norwegian data protection regulations.
