# Supplementary material for: Cryptogenic stroke in women: impact of insertable cardiac monitoring in the STROKEWISE cohort study
Source: Europace. 2026 Mar 23;28(4):euag056. doi: 10.1093/europace/euag056 (PMC13122606; doi:10.1093/europace/euag056)
Supplement: euag056_Supplementary_Data [file euag056_supplementary_data.docx]

## Supplementary Table 1. Univariable predictors of recurrent ischaemic stroke (excluding atrial fibrillation)

|  | Recurrent (n=32) | No recurrent stroke (n=441) | OR (95% CI) | P-value |
| --- | --- | --- | --- | --- |
| Hypertension, n (%) | 26 (81) | 265 (60) | 2.88 (1.17–7.10) | 0.023† |
| Prior ischaemic stroke (≥2), n (%) | 8 (25) | 54 (12) | 2.39 (0.99–5.77) | 0.053 |
| Diabetes mellitus, n (%) | 8 (25) | 59 (13) | 2.15 (0.90–5.15) | 0.109 |
| Prior myocardial infarction, n (%) | 4 (13) | 22 (5) | 2.90 (0.89–9.42) | 0.090 |
| Creatinine (per 10 µmol/L) | – | – | 1.06 (1.01–1.11) | 0.021† |
| Troponin T (per ng/L) | – | – | 1.03 (1.00–1.06) | 0.071 |

*Odds ratios derived from univariable logistic regression analyses. Numbers may vary slightly due to missing data. OR, odds ratio; CI, confidence interval. †Two-sided P<0.05.*

## Supplementary Table 2. Multivariable logistic regression analysis for recurrent ischaemic stroke

|  | Adjusted OR | 95% CI | P-value |
| --- | --- | --- | --- |
| Atrial fibrillation | 1.14 | 0.50–2.59 | 0.761 |
| Hypertension | 3.05 | 1.13–8.24 | 0.028 |
| Creatinine (per 10 µmol/L) | 1.04 | 0.97–1.11 | 0.244 |
| Age (per year) | 1.02 | 0.98–1.05 | 0.395 |

*Multivariable logistic regression adjusted for atrial fibrillation, age and renal function. OR, odds ratio; CI, confidence interval.*

## Supplementary Table 3. Exploratory, unadjusted analyses of clinical outcomes by hospital

|  | ICM (n=262) | Control (n=213) | Total (N=475) | OR (95% CI) | P-value |
| --- | --- | --- | --- | --- | --- |
| Recurrent ischaemic stroke (all patients) | 18 (6.9) | 14 (6.6) | 32 (6.7) | 1.04 (0.50–2.16) | 0.898 |
| Recurrent stroke, no AF detected | 12/169 (7.1) | 11/194 (5.7) | 23/363 (6.3) | 1.27 (0.54–3.01) | 0.668 |
| AF among patients with recurrent stroke | 6/18 (33.3) | 3/14 (21.4) | 9/32 (28.1) | 1.83 (0.36–9.38) | 0.694 |
| GI bleeding (entire cohort) | 6 (2.3) | 14 (6.6) | 20 (4.2) | 0.33 (0.12–0.86) | 0.021 |
| GI bleeding, OAC during follow-up | 3/109 (2.8) | 7/75 (9.3) | 10/184 (5.4) | 0.27 (0.07–1.05) | 0.093 |
| GI bleeding, no OAC during follow-up | 3/153 (2.0) | 7/138 (5.1) | 10/291 (3.4) | 0.38 (0.10–1.48) | 0.146 |
| Symptomatic intracranial haemorrhage (entire cohort) | 2 (0.8) | 6 (2.8) | 8 (1.7) | 0.27 (0.05–1.34) | 0.084 |
| Symptomatic intracranial haemorrhage, OAC during follow-up | 2/109 (1.8) | 5/75 (6.7) | 7/184 (3.8) | 0.26 (0.05–1.38) | 0.123 |
| Symptomatic intracranial haemorrhage and empirical OAC at discharge | — | 4/55 (7.3) | — | — | 0.020 |
| Total mortality (entire cohort) | 39 (14.9) | 68 (31.9) | 107 (22.5) | 0.37 (0.23–0.59) | <0.001 |
| Mortality, OAC during follow-up | 19/109 (17.4) | 20/75 (26.7) | 39/184 (21.2) | 0.58 (0.29–1.18) | 0.145 |
| Empirical OAC at discharge | 0/262 (0.0) | 55/213 (25.8) | 55/475 (11.6) | — | — |

*Exploratory, unadjusted χ² and Fisher’s exact tests. No multivariable adjustment performed due to limited event numbers. AF, atrial fibrillation; ICM, insertable cardiac monitor; OAC, oral anticoagulation.*

## Supplementary Table 4. Recurrent ischaemic stroke according to hospital and oral anticoagulation (OAC) status

|  | ICM, n/N (%) | Control, n/N (%) | P-value |
| --- | --- | --- | --- |
| Recurrent ischaemic stroke | 18/262 (6.9) | 14/213 (6.6) | 0.898 |
| Never on OAC during follow-up | 13/153 (8.5) | 4/138 (2.9) | 0.042† |
| Any OAC during follow-up | 5/109 (4.6) | 10/75 (13.3) | 0.033† |

*Exploratory, unadjusted χ² tests. No multivariable adjustment performed due to limited event numbers. ICM, insertable cardiac monitor; OAC, oral anticoagulation. †Two-sided P<0.05.*
